# Cell-Free DNA Levels During the First Hours After Liver Transplantation: A Key Biomarker for Patient Survival and Outcomes

**DOI:** 10.3390/jcm14238400

**Published:** 2025-11-27

**Authors:** Hada C. Macher, José L. Rubio-Prieto, Noelia García-Fernández, Patrocinio Molinero, Miguel A. Gómez-Bravo, Juan M. Guerrero, Gonzalo Suárez-Artacho, Amalia Rubio

**Affiliations:** 1 Department of Clinical Biochemistry, Instituto de Investigaciones Biomédicas de Sevilla, IBIS, University of Seville, HUVR, Junta de Andalucía, CSIC, 41013 Seville, Spain; hadacmacher@icloud.com (H.C.M.); jose.rubio.prieto.sspa@juntadeandalucia.es (J.L.R.-P.); noegarfe1@gmail.com (N.G.-F.); guerrero@us.es (J.M.G.); 2 Department of Medical Biochemistry and Molecular Biology and Immunology, Instituto de Investigaciones Biomédicas de Sevilla, IBIS, University of Seville, HUVR, Junta de Andalucía, CSIC, 41013 Seville, Spain; molinero@us.es; 3 Hepatobiliary and Liver Transplantation Unit, Virgen del Rocío University Hospital, 41013 Seville, Spain; miagbravo@us.es (M.A.G.-B.); gsartacho@gmail.com (G.S.-A.)

**Keywords:** cell-free DNA, liver transplantation, war and cold ischemia, patient outcome

## Abstract

(1) **Background**: We propose that cfDNA levels may serve as a valuable biomarker for monitoring the progress of liver transplant recipients, reflecting the quality of the donated organ, and predicting patient prognosis and survival. Thus, we analyzed the relationship between total cfDNA levels during the first 48 h post-transplantation, ischemia–reperfusion injury, and patient outcomes. (2) **Methods**: cfDNA quantification was applied to 115 liver transplant patients using real-time quantitative PCR at the time of transplantation (during reperfusion) and throughout the first month post-transplantation. (3) **Results**: Significantly higher early cfDNA levels were observed in patients who suffered liver damage or post-transplantation complications during the first month. High cfDNA levels were also associated with prolonged ICU stays and reduced survival. Kaplan–Meier analysis revealed a significantly lower survival rate in patients with elevated cfDNA. CRP levels were elevated and significantly correlated with cfDNA values. Regarding organ preservation prior to transplantation, prolonged cold and warm ischemia times were significantly associated with high cfDNA levels in the early hours post-transplantation. (4) **Conclusions**: Elevated cfDNA levels in the early hours following liver transplantation are associated with poorer patient outcomes. Therefore, determining total cfDNA levels post-transplantation may be a valuable tool for patient management and early intervention.

## 1. Introduction

Liver transplantation has advanced significantly in recent years and is now considered the optimal treatment for various liver diseases. Not only has the liver transplantation rate increased substantially in many countries, but the long-term survival rate of liver transplant patients has clearly increased, contributing to a generally good quality of life [1].

Preventing and managing complications associated with this procedure is critical to achieving successful outcomes. However, long-term graft failure still occurs in some patients due to organ rejection or factors related to either the recipient or the donated organ. The most common complications during the first year, aside from acute rejection, include vascular and biliary issues, primary graft dysfunction, and recurrence of hepatitis C virus (HCV), all of which compromise graft function and patient survival [1,2].

Cell death resulting from necrosis or apoptosis is widely recognized as a significant source of DNA release into plasma. Changes in cell-free DNA (cfDNA) levels have been identified as a promising biomarker in various clinical settings, including transplant monitoring. Donor-specific cfDNA has shown potential in predicting organ damage or rejection [3,4]. Moreover, it has been reported that a range of complications—whether related to the donated organ or general patient health—are associated with increased cfDNA levels. Elevated cfDNA during the initial days post-transplantation may indicate a worse prognosis and has been linked to decreased survival rates [5].

The quality of the donated liver is a critical determinant of transplant success. Liver transplantation involves a period of ischemia, both cold and warm. Upon restoration of blood flow, tissue injury may worsen and, in some cases, become irreversible. This clinical condition is referred to as ischemia–reperfusion injury (IRI). IRI is a major contributor to post-surgical liver dysfunction [6]. Early-stage organ failure due to IRI occurs in approximately 10% of cases and constitutes a significant risk factor for both acute and chronic liver transplant rejection [7].

Research suggests that IRI can lead to cell death through apoptosis or necrosis. Several animal model studies have demonstrated a strong association between IRI and liver cell apoptosis. The findings suggest that apoptosis of endothelial cells and hepatocytes plays a key role in cell death following IRI [8]. A retrospective study involving liver transplant patients also showed that cell apoptosis is likely associated with reperfusion injury [9]. Based on these findings, it can be hypothesized that elevated cfDNA levels result from apoptosis induced by IRI. Factors such as liver preservation procedures and the duration of cold and warm ischemia are critical to determining graft viability and transplant success. Extended ischemia time increases the risk of IRI once blood flow is restored to the transplanted organ [7]. Therefore, minimizing cold ischemia time is essential to improving transplantation outcomes.

Post-transplant cfDNA levels may thus serve as a valuable biomarker for monitoring patient recovery, assessing donor organ quality, and predicting patient prognosis and survival. This study aimed to investigate the relationship between total cfDNA levels during the first 48 h post-transplantation, ischemia–reperfusion injury, and patient outcomes. Our findings indicate that early cfDNA levels are associated with organ preservation conditions, measured through warm and cold ischemia times, and correlate with poor post-transplant patient outcomes.

## 2. Materials and Methods

### 2.1. Study Subjects

All consecutive patients who underwent orthotopic liver transplantation at Virgen del Rocío University Hospital (Seville, Spain) over a four-year period were invited to participate in the study for cfDNA monitoring. Each patient was clinically evaluated for at least two years following inclusion. After being placed on a waiting list, candidates were informed about the study unless their clinical condition prevented them from understanding its purpose. Upon agreement, written informed consent was obtained prior to inclusion.

Eligible participants met the following inclusion criteria: (a) age ≥ 18 years and (b) provision of signed informed consent. During transplantation, two central venous blood samples were collected: a basal sample at the onset of anesthesia induction (anhepatic phase) and another 15 min after graft reperfusion.

Patients with missing or invalid serum samples at the time of transplantation or with less than one week of postoperative follow-up were excluded. A total of 115 liver transplant recipients were finally included in the study. The general characteristics of the study population are summarized in Table 1.

### 2.2. Patient Surveillance and Treatment

Patients were clinically and biologically evaluated during their stay in the Intensive Care Unit (ICU) and subsequently in the general ward. Peripheral venous blood samples were collected daily during the first three postoperative days and every 2–3 days thereafter, using Vacuette^®^ 9 mL Z Serum Separator Clot Activator tubes (Greiner Bio-One, Kremsmünster, Austria). Serum cfDNA levels, as well as standard biochemical, hematological, and coagulation parameters, were monitored, and immunosuppressive drug concentrations were determined.

For cfDNA analysis, 10 mL blood samples were centrifuged for 5 min at 3500 rpm at room temperature within 6 h of collection. Serum was then aliquoted and stored at −80 °C until analysis. After hospital discharge, patients were followed up at scheduled outpatient visits, during which blood samples were obtained. DNA extraction from frozen samples was performed within a maximum storage period of 6 months.

Immunosuppressive therapy consisted of tacrolimus, mycophenolate mofetil, and steroids. Target blood levels of calcineurin inhibitors were maintained at approximately 7 ng/mL during the first month, 5–7 ng/mL throughout the first year, and below 5 ng/mL thereafter. In patients with chronic renal dysfunction, an induction protocol with basiliximab combined with delayed, reduced-dose tacrolimus, mycophenolate mofetil, and steroids was used.

### 2.3. DNA Extraction from Tissue and Serum Samples

DNA was extracted from 400 μL of serum using the automated MagNA Pure Compact Instrument (Roche Diagnostics, Basel, Switzerland) and the MagNA Pure Compact Nucleic Acid Isolation Kit I, following the protocol “Total NA Plasma 100–400 V3.1”, in a final elution volume of 50 μL, and stored at −80 °C until qPCR analysis. Quantification of nucleic acids after isolation was performed using Qubit 3.0 fluorometry (Thermo Fisher Scientific, Waltham, MA, USA) according to the manufacturer’s instructions.

### 2.4. Quantification of cfDNA by Real-Time Quantitative PCR (qPCR)

The patients’ cfDNA was quantified by amplification of the β-globin gene using a LightCycler 480 Real-Time PCR System (Roche Diagnostics, Basel, Switzerland). For each reaction, 5 μL of extracted DNA was amplified in a final volume of 20 μL containing 200 nM of each primer and 100 nM of the probe, using the LC480 Probes Master Kit (Roche Diagnostics). The 2× concentrated master mix is optimized for a fixed MgCl_2_ concentration compatible with nearly all primer sets, eliminating the need for further adjustment.

qPCR amplification was performed under the following cycling conditions: 95 °C for 5 s and 61 °C for 20 s, for a total of 40 cycles. Standard calibration curves were generated using serial dilutions of human genomic DNA (Roche Diagnostics). The slope of the calibration curves ranged from 3.34 to 3.85, corresponding to a PCR efficiency between 1.81 and 1.99. The lowest standard concentration provided a detection limit of 1.5 Genomic Equivalents (GE)/mL of serum. The error value (mean squared error of the regression fit) was consistently below 0.2 [0.0004–0.01], indicating high quantification accuracy. Primer and probe sequences, fragment length, and annealing temperatures were previously described [10].

All samples from each patient were analyzed in a single run, and each was assayed in duplicate. A no-template control was included in every run to monitor potential contamination. Final cfDNA concentrations were calculated according to the following formula (*GE*: Genomic Equivalent; *V_DNA_*: total volume of cfDNA after serum extraction; *V_PCR_*: PCR sample volume; *V_ext_*: extracted serum volume).

$$c=GE\times \frac{V_{DNA}}{V_{PCR}}\times \frac{1}{V_{ext}}$$

### 2.5. Statistical Analysis

IBM SPSS version 26 was used for all statistical analyses. Comparisons between two continuous variables (shown by median and range) were performed using the *t*-test for normal distribution and the Mann–Whitney U-test for non-normal distribution. Multiple comparison analysis was performed using ANOVA for normal distribution and the Friedman test for non-normal distribution. Comparisons between related variables were performed using the Wilcoxon test. Correlations between variables were performed using the Spearman test.

### 2.6. Ethics Statement

This research complies with all relevant national regulations and institutional policies. Ethical approval for the study protocol was obtained from the Medical Research Ethics Board of the Virgen del Rocío University Hospital (Seville, Spain). All patients included read and signed an informed consent form to participate in the study. The clinical investigation was conducted according to the principles expressed in the Declaration of Helsinki.

## 3. Results

### 3.1. Characteristics of the Patients

The general characteristics of the study population and the comparison between fully stable patients (73%) and those who developed any form of hepatic injury during the first month of follow-up (27%) are presented in Table 1. The median age of the recipients was 56 years, while the median donor age was higher (62 years), with 16% of donors being older than 75 years. Eleven patients remained in the ICU for several days prior to TX, although only two stayed for more than 4 days (34 and 58 days, respectively). After surgery, all patients were admitted to the ICU, where the length of stay varied widely depending on clinical progression (range: 3–55 days).

Patients without (stable patients) and with any kind of hepatic damage, including rejection (14 patients) and primary allograft dysfunction (11 patients), were compared. Both age and total hospitalization time differed significantly between the two groups. Stable patients were generally older, whereas individuals with hepatic damage exhibited a significantly longer total hospitalization time. Furthermore, the liver damage group presented a significantly higher proportion of patients who stayed at the ICU prior to TX. Although the warm ischemia median was 31 min for both groups, three patients who presented a long warm ischemia time (1 h) showed hepatic damage. In the same way, the three patients with a significantly high period of cold ischemia (9 h) belong to this group. Patients with poor outcomes during the first days after TX, with a long period at ICU, also experienced a longer hospitalization period (positive correlation of time at ICU and hospitalization days, Spearman test; *p* < 0.0001).

Major liver disease before transplantation was alcoholic cirrhosis and hepatocellular carcinoma, either independently or both present. No significant differences were observed between patients with and without hepatic damage. In the same way, the Model for End-Stage Liver Disease (MELD) score was quite variable and similar between both groups analyzed. The CHILD score was not clinically analyzed for all patients. Of the patients analyzed, most were B (54%), followed by C (24%) and A (21%).

Graft rejection during the completed follow-up was reported in 21 patients (18%), and the rejection occurred during the first month after transplantation in 14 cases (12.1%) (Table 1). In most instances, rejection episodes were controlled by adjusting the immunosuppressive regimen. Table 2 summarizes the comparison between patients with and without rejection during the first month after TX. Despite the low number of patients included in the rejection group, we found similar results. Thus, patient age and hospitalization time were also statistically different between groups. Although differences in the proportion of patients who stayed at the ICU prior to TX were observed, these differences did not reach statistical significance.

A total of 11 patients (9.5%) died within three years following transplantation. One patient died early after acute graft rejection (day 420), while the remaining deaths were due to diverse causes over a period ranging from 4 days to 34 months post-TX. Detailed causes and timing of death are provided in Supplementary Table S1.

### 3.2. Patient Outcomes and cfDNA Levels

**Figure 1:**
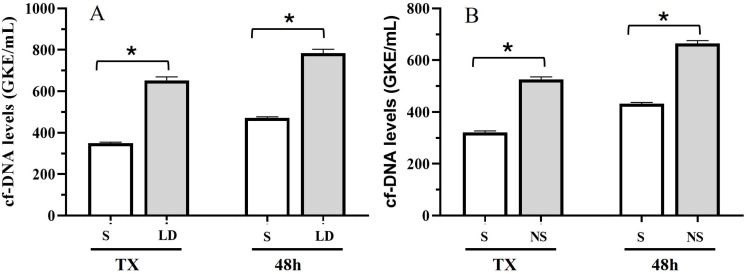
cfDNA levels at TX and during first 48 h for (**A**) stable patients (S) and patients with liver damage (LD); and (**B**) stable patients and individuals with any complication (NS), during the first month of follow-up. (*t* test (*) *p* < 0.05).

cfDNA during the first 48 h was quite variable among patients (median at TX, 428.54; range, [15.27–3447]). Comparing patients with or without liver damage during the first month of follow-up, we observed significantly higher cfDNA levels at TX and during the first 48 h in patients with liver damage. Furthermore, cfDNA levels were significantly higher compared with stable patients and individuals with complications during the first month of follow-up (Figure 1A,B; *t*-test (*), *p* < 0.05).

**Figure 2:**
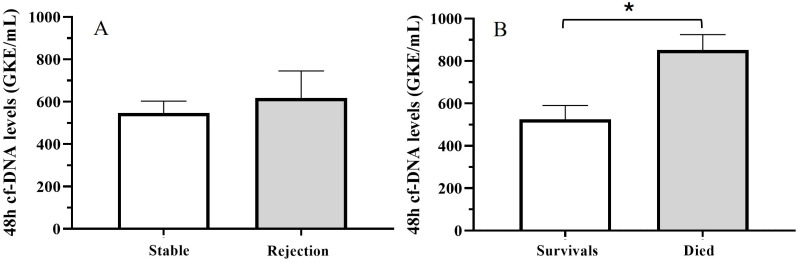
Mean 48 h cfDNA levels for (**A**) stable patients and patients who suffered rejection during the first month of follow-up; (**B**) survival patients and patients who died during the first three years after TX (*t* test (*) *p* < 0.05).

Only 14 patients suffered a rejection during the first month of follow-up. Although cfDNA values during the first 48 h were higher in this group of patients, the differences did not reach statistical significance, probably due to the low number of patients in the rejection group. Conversely, we found statistical differences comparing cfDNA levels in 11 patients who died during the first three years and surviving patients (Figure 2A,B; Mann–Whitney U-test, *p* < 0.05).

**Figure 3:**
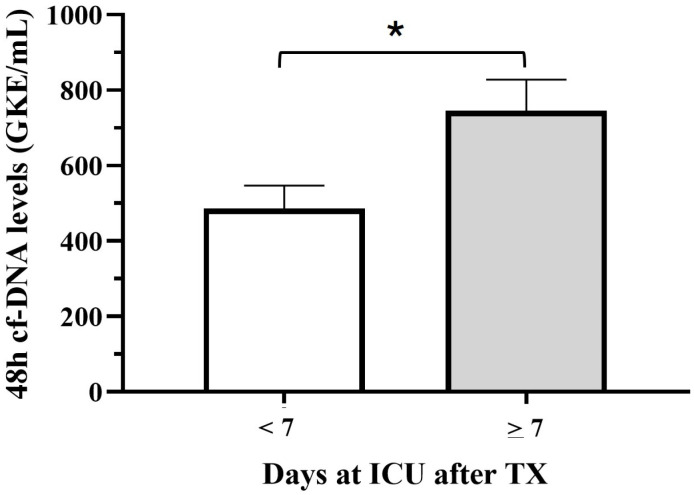
Comparison of mean 48 h cfDNA levels according to the period of stay at ICU (*t* test (*) *p* < 0.05).

Furthermore, we observed a significant correlation between the number of days at the ICU after transplantation and cfDNA levels at TX (Spearman correlation, *p* = 0.006). Thus, mean cfDNA levels at TX and during the first 48 h were higher for patients with more than 1 week of stay at the ICU (745.3 + 148 and 717.19 + 167) than for patients with a shorter period at the ICU (486.11 + 66 and 322.94 + 43.2) (*t*-Test, *p* < 0.05) (Figure 3).

**Figure 4:**
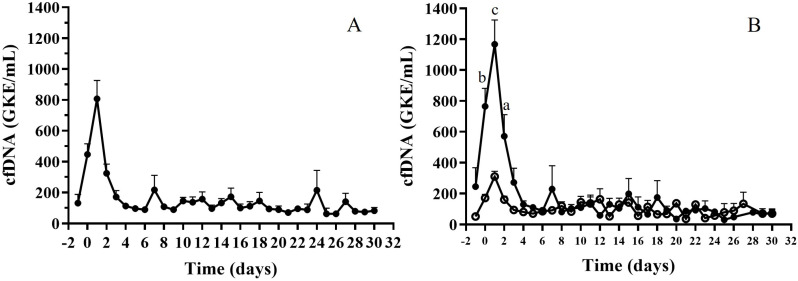
(**A**) cfDNA levels during the first month post TX; (**B**) cfDNA values after stratification of patients with high (HcfDNA; black circles) or low (LcfDNA; white circles) initial values. ANOVA test, (a) *p* < 0.005; (b) *p* < 0.0001; (c) *p* = 0.0002.

Figure 4A shows cfDNA during the first month of follow-up. cfDNA significantly increased at TX and during the first 24 h, decreasing with different profiles depending on the patient’s progress. Patients were stratified according to initial cfDNA values (HcfDNA: levels over 600 ng/mL; LcfDNA: levels below 600 ng/mL). We observed significant differences between groups related to liver damage, with a higher proportion of patients with liver damage in the HcfDNA group (38% vs. 21%; chi-squared test, *p* < 0.05). In addition, a higher proportion of patients died during the first years in the HcfDNA group (71% vs. 29%; chi-squared test; *p* < 0.05). Even though the initial mean cfDNA level was 3.5 times higher for HcfDNA, cfDNA level evolution only differed during the first days, with statistically different values at TX, day one, and two (ANOVA test: *p* = 0.0002, *p* < 0.0001, and *p* < 0.005, respectively). Subsequently, cfDNA levels were similar between these two groups (Figure 4B).

**Figure 5:**
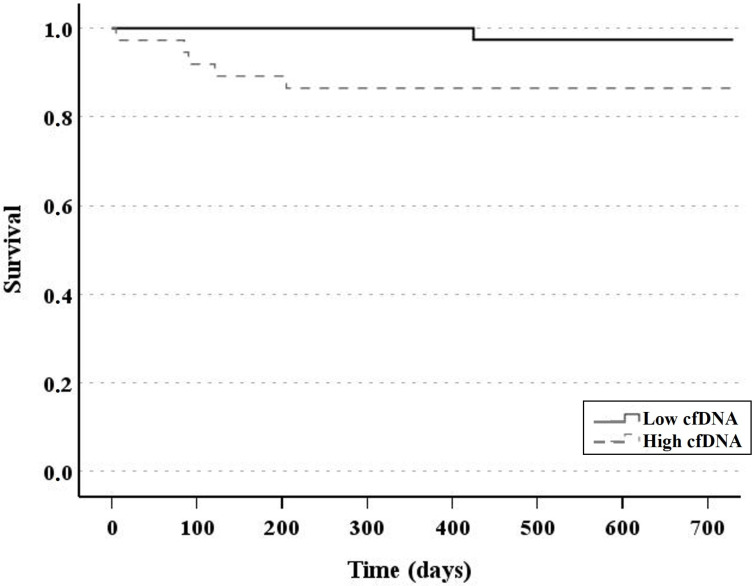
Two years survival curves for both groups of patients, HcfDNA (dash line) and LcfDNA (continuous line). Kaplan–Meier test; *p* < 0.05.

Three-year survival was also analyzed in relation to cfDNA levels. Figure 5 shows the Kaplan–Meier curves for both groups, HcfDNA and LcfDNA. We observed a significantly high survival rate for LcfDNA patients (97% versus 86%, *p* < 0.05). Furthermore, among the 11 patients who died, five deaths occurred during the first year of follow-up, and all belonged to the HcfDNA group.

### 3.3. Relationship Between cfDNA and Biochemical Biomarkers

**Figure 6:**
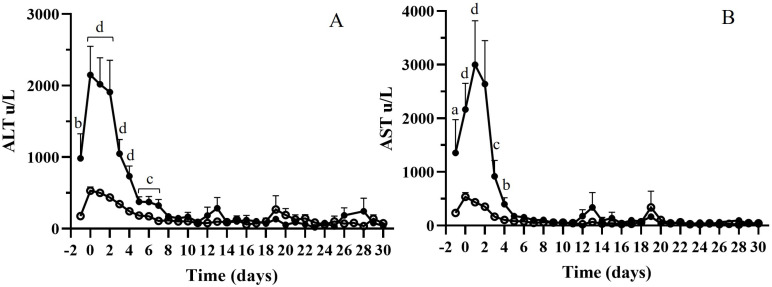
(**A**) alanine aminotransferase (ALT) and (**B**) aspartate aminotransferase (AST) levels during the first 30 days for the two groups of patients, HcfDNA (black circles) and LcfDNA (white circles). ANOVA test, (a) *p* < 0.05; (b) *p* < 0.005; (c) *p* < 0.001; (d) *p* = 0.0001.

The relationship between cfDNA and classical liver function biomarkers was analyzed. The evolution of alanine aminotransferase (ALT) and aspartate aminotransferase (AST) levels during the first 30 days showed a similar profile to that of cfDNA levels and opposite to that of gamma-glutamyltransferase (gamma-GT) levels (Supplementary Figure S1). Table 3 shows the correlation of cfDNA with ALT, AST, gamma-GT, and bilirubin levels during the first two weeks of follow-up. In both analyses, the total population and the stable and liver damage groups showed a significant correlation between ALT, AST, and cfDNA levels (Spearman test, Table 3). Stable patients also showed a significant correlation with gamma-GT values. No correlation between bilirubin and cfDNA levels was observed. Liver enzyme changes differed based on cfDNA levels. Figure 6 shows ALT and AST levels for the two groups, and significant differences were observed during the first days of follow-up between these two groups (ANOVA test).

### 3.4. Relationship Between cfDNA and Immunological Parameters

**Figure 7:**
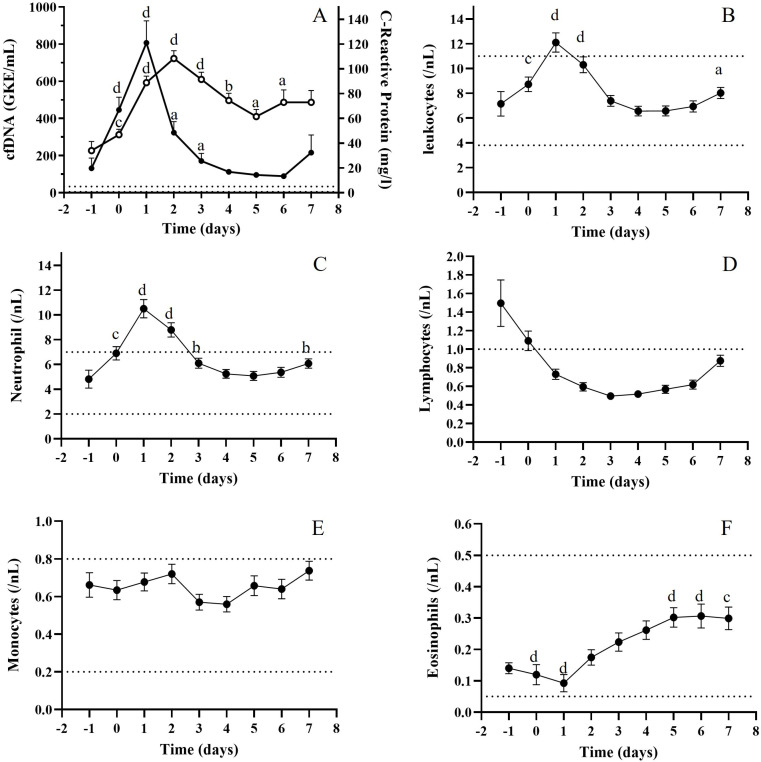
(**A**) C-reactive protein (CRP) levels and cfDNA levels during the first week after TX; (**B**) total leukocytes; (**C**) neutrophils; (**D**) lymphocytes; (**E**) monocytes and (**F**) eosinophils levels during the first week after TX. Wilcoxon test, (a) *p* < 0.01; (b) *p* < 0.005; (c) *p* < 0.001; (d) *p* < 0.0001.

C-reactive protein (CRP) levels were considerably elevated during the first weeks of follow-up compared with values before TX (Friedman test, *p* < 0.01), with significant variations from day 0 after TX (Wilcoxon test). We observed a peak at day 2 (Figure 7A), delayed 24 h from the cfDNA peak, that decreased after this point. However, it did not reach basal levels during the first weeks after TX.

cfDNA correlated with CRP levels during the first two weeks of follow-up (Spearman test, *p* < 0.001). Furthermore, cfDNA levels at TX correlated with CRP at the moment of transplantation and 48 h later (Spearman test *p* < 0.05). cfDNA levels previous to TX also correlated with CRP at this time point and at TX (Spearman test, *p* < 0.005 and *p* < 0.05, respectively).

As this inflammation marker was highly elevated during the first week, we explored the evolution of the different immune cell populations during this period. Figure 7B–F shows the total leukocyte, neutrophil, lymphocyte, and eosinophil levels during the first week after TX. Leukocyte levels were significantly elevated during the first 48 h. Of these, neutrophils were the most abundant white cells, with significant differences from pre-TX to day 3 and a high peak matching with the cfDNA peak (Wilcoxon test). Patients showed a clear, significant decrease in lymphocyte numbers after TX, probably due to an immunosuppressor treatment (Figure 7D). Low eosinophil numbers decreased during the first 24 h and significantly increased after this point, but always within the normal reference range (Figure 7E). CRP levels and white cell counts were evaluated when comparing the two groups. We observed significantly higher lymphocyte levels just before TX and at TX in the HcfDNA group (Wilcoxon test, *p* < 0.005 and *p* < 0.05, respectively) and higher neutrophil numbers, but this was only significantly different before TX (Wilcoxon test, *p* < 0.005).

### 3.5. Relationship Between cfDNA and Ischemia Time

**Figure 8:**
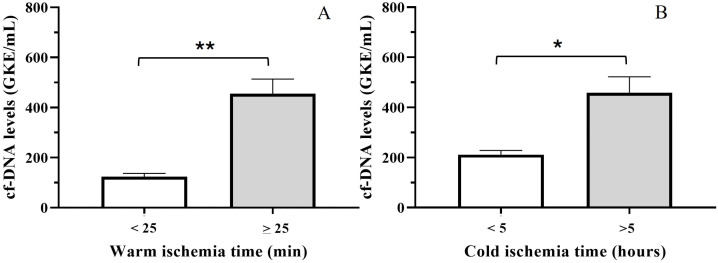
cfDNA levels at TX according to hepatic ischemia–reperfusion injury. (**A**) Warm ischemia time and (**B**) cold ischemia time. *t*-test, (*) *p* < 0.005 and (**) *p* < 0.0001.

cfDNA levels at TX were analyzed in relation to hepatic ischemia–reperfusion injury. As most patients had an adequate warm and cold ischemia time, they were stratified as having high-quality preserved organs (warm ischemia time < 25 min; cold ischemia time < 5 h). We observed a significant relationship between both cold and warm ischemia time. Thus, a longer ischemia time was significantly associated with high cfDNA levels (Figure 8A,B; *t*-test, *p* < 0.0001 and *p* < 0.005). Warm ischemia times were more strongly related to cfDNA levels, and thus, a significant correlation between warm ischemia time and cfDNA levels during the first 48 h was observed (Spearman test, *p* = 0.005). In addition, HcfDNA patients presented a longer period of warm ischemia (Mann–Whitney U-test, *p* < 0.05).

The group with a long period of warm ischemia showed moderate levels of cfDNA. We analyzed the percentage of patients suffering liver damage or any complication in those suffering from long periods of warm ischemia in relation to cfDNA. Comparing patients with LcfDNA, HcfDNA, and liver damage, we observed that only 3% of LcfDNA patients presented liver damage compared with 44% of HcfDNA patients (chi-squared test, *p* < 0.001). Similar results were observed for general patient complications (chi-squared test, *p* = 0.05). No significant differences were found when a shorter period of warm ischemia was studied.

## 4. Discussion

The results show that high levels of total cfDNA during the first hours after surgery in liver transplant patients are associated with poor outcomes post-transplantation. Furthermore, long periods of either cold or warm ischemia are also related to high levels of cfDNA and may influence graft dysfunction, compromising transplantation success. We propose the quantification of total cfDNA levels during the first hours after transplantation as a useful tool for liver transplant patient management.

The quantification of total cfDNA has emerged as a promising biomarker for assessing overall tissue injury and patient deterioration [5,11]. Although cfDNA is detectable under physiological conditions, its concentration rises markedly in various pathological states. As previously reported, cfDNA levels measured in samples collected during surgery, specifically at the moment of organ reperfusion, show a pronounced transient increase that gradually declines as the patient’s condition stabilizes [4,12]. However, cfDNA levels are quite variable during the first hours and may determine the patient’s outcome after transplantation. We have observed that after stratifying patients according to initial cfDNA levels, the evolution profile differed only during the first days, with similar values after 72 h post-transplant. These high variations during this period may be related to patient evolution. We tried to establish a relationship between initial cfDNA levels and patient outcome. Results from a preliminary study with fewer patients showed that during the first 72 h, a different decreasing profile can be observed when comparing patients with or without post-transplantation complications [13]. Additionally, we described a significantly different cfDNA decay pattern when comparing heart transplant patients with good outcomes and patients with complications [10].

Our results show significantly higher levels of cfDNA during the first 48 h in patients with complications during the first month, either directly associated with liver damage or not. This worse outcome was reflected in the longer ICU stay for patients with high levels of cfDNA. Agreeing with our results, Blasi et al. [14] have described higher levels of cfDNA in acutely ill patients with cirrhosis who suffered liver failure.

Furthermore, cfDNA levels seem to be related to long-term patient outcomes, as higher initial cfDNA levels were observed in the 11 patients who died during the first three years. Other groups have also described higher levels of cfDNA in patients who died after short [14] or longer follow-up [5] after transplantation. In this way, patients with high cfDNA levels suffer a higher incidence of portal hepatitis and show inferior survival rates due to liver abscess and sepsis [5]. We also observed a significantly lower survival rate for HcfDNA patients, and all deaths during the first year of follow-up occurred in this group.

It has been hypothesized that high cfDNA may reflect cellular trauma and inflammation during surgery, being suggestive of a surgical stress marker [15]. Furthermore, elevated cfDNA values may reflect ischemia–reperfusion injury during liver transplantation. It has been well described that patients with elevated cfDNA levels, due to early unresolving allograft injury, are at risk of subsequent liver dysfunction. The high survival rate for LcfDNA patients observed in this study supports this information.

Liver function biomarkers—ALT, AST, gamma-GT, and bilirubin—also provide key evidence of liver damage. We observed that ALT and AST levels were also significantly related to cfDNA levels. Moreover, the profile level of both aminotransferases, ALT and AST, was quite different according to cfDNA levels, with significantly higher levels in the HcfDNA group during the first days. However, these liver function biomarkers are not always clearly elevated after transplantation complications. We have previously described a case of successful liver transplantation where the patient suffered sustained biliary complications during early evolution that finally ended in cholestasis. After bilirubin, ALT, and AST level normalization, the patient was discharged. However, she was admitted again to the ICU because of sepsis caused by biliary obstruction, developed a multiorgan failure, and finally died. A retrospective determination of cfDNA showed a five-time increase at discharge, coincident with normalized bilirubin, ALT, and AST levels, highlighting the key role of cfDNA as a graft complication biomarker [13]. These results suggest that total cfDNA could serve as a prognostic marker for patient outcomes and allow clinicians to carry out early interventions.

It has been proposed that DNA fragments released from apoptotic or necrotic cells contribute to the activation of pro-inflammatory pathways [5,16,17]. Some authors further suggest that the increase in cfDNA is not merely a by-product of cell death but may play an active physiological and pathological role in the modulation of inflammation and autoimmunity [18]. In this context, histones and cfDNA within nucleosomes function as damage-associated molecular patterns (DAMPs), acting as potent pro-inflammatory stimuli that activate the innate immune response [19]. Moreover, the inflammatory activation of Kupffer cells, which are abundant in the liver and represent a central component of the innate immune system, has been proposed as a key mechanism contributing to graft dysfunction [20].

We observed remarkably high CRP levels during the first week of follow-up. CRP values during the first two weeks significantly correlated with cfDNA values. Median basal level pre-surgery was above the superior reference value and continued increasing after transplantation, reaching a peak value at day two (20-fold superior reference value). The peak value was 24 h delayed from the cfDNA peak, and we speculate that high levels of cfDNA may trigger CRP levels. Supporting this, we found that cfDNA levels at TX correlated with CRP at the moment of transplantation and two days later.

Leukocyte numbers significantly increased during the first 48 h. However, only neutrophils presented levels above the superior reference values with a peak value at day one, coinciding with the cfDNA peak. By contrast, lymphocyte numbers decreased during the follow-up below the lower reference value, probably due to the immunosuppressor treatment. Despite lymphocyte cells, no differences were observed comparing patients from the HcfDNA and LcfDNA groups when white cell counts were analyzed. We observed higher neutrophil numbers in the HcfDNA group, but they did not reach statistical significance. This suggests that the molecular microenvironment is more important than the number of leukocytes.

The quality of the donor liver is a critical determinant of transplant success. Timely restoration of oxygen delivery is essential for rescuing ischemic tissue; however, reperfusion of blood to an ischemic organ represents a paradoxically harmful event that triggers IRI [21]. At the moment of organ reperfusion, total cfDNA levels increased markedly, although substantial interindividual variability was observed among patients. IRI has been recognized as a major contributor to postoperative liver dysfunction [6], increasing the risk of graft failure [7]. Early organ failure resulting from IRI may affect up to 10% of liver transplant recipients and can readily progress to acute or chronic rejection [22]. Necrotic and apoptotic processes after IRI may increase cfDNA levels released by hepatocytes. As liver preservation procedures, including the duration of warm and cold ischemia, are critical for transplantation success, we speculate that a shorter ischemia period could decrease the risk of IRI and, therefore, result in diminished cfDNA levels. We observed that a longer period of both cold and warm ischemia was significantly associated with high cfDNA levels during the first hours after transplantation. Among these, warm ischemia time was more strongly related to cfDNA levels, with a significant correlation during the first 48 h. Warm ischemia exerts deleterious effects on the liver graft and should, therefore, be minimized during surgery. By contrast, some authors have reported no association between warm ischemia duration and cfDNA peaks during liver transplantation, although these findings may have been constrained by limited sample sizes [23]. Even though a longer period of warm ischemia was associated with high cfDNA levels, a group of patients showed moderate levels of cfDNA and a long warm ischemia time. Interestingly, we found fewer graft injuries in these patients than in the HcfDNA group. Thus, cfDNA levels at TX seem to be a strong biomarker that reflects the grade of liver damage; therefore, we postulate that this should be measured as a progression biomarker for early intervention.

cfDNA is a promising biomarker for assessing patient damage and deterioration, with levels rising in pathological states. Our results show that total cfDNA level quantification during the first hours after transplantation may be an important tool for patient management. On the other hand, the relationship observed between high levels of initial cfDNA levels and ischemia time also supports the importance of cfDNA determination during the first hours after TX. Furthermore, we found that patients with long ischemia times and low cfDNA had better outcomes than those with high cfDNA levels, supporting the idea that initial cfDNA determination is an important biomarker that can guide early intervention. Furthermore, we previously found that cfDNA may be elevated during the follow-up due to several complications that do not alter liver function markers. This result suggests the importance of measuring cfDNA, not only at TX but also during hospitalization and before discharge.

## 5. Conclusions

In conclusion, we propose quantifying cfDNA levels during the first hours after transplantation to improve the early clinical management of patients. Our results suggest that elevated cfDNA levels in the first hours after liver transplantation are linked to poor patient outcomes. On the other hand, long periods of both cold and warm ischemia may determine liver dysfunction, compromising transplantation success. The association of long ischemia time and elevated cfDNA reinforces the initial cfDNA determination as an approach for early intervention, modifying, for example, immunosuppression treatment before the occurrence of adverse events after liver transplantation. This approach may enable clinicians to take an active rather than reactive approach to patient management.

## Figures and Tables

**Table 1:** General characteristics of the patients. Comparison between stable patients and patients undergoing hepatic damage.

		Total (*n* = 115)	Stable (*n* = 84)	Hepatic Damage (*n* = 31)	*p*
Age (years)		56 [18–71]	57 [22–71]	51 [18–68]	**0.01**
Sex (male)		62 (53.4%)	45 (52.9%)	17 (54.8%)	0.51
Donnor age		62 [14–84]	63 [17–84]	58 [14–82]	0.31
Warm Ischemia Time (min)		31 [22–60]	31 [23–70]	31 [22–60]	0.85
Cold Ischemia Time (min)		385 [250–575]	385 [270–551]	377 [250–575]	0.25
Previous ICU		11 (9.5%)	5 (5.8%)	6 (19.4%)	**0.04**
Previous ICU time (days)		3 [1–58]	3 [2–58]	2 [1–34]	0.29
ICU post-TX (days)		6 [3–55]	6 [3–55]	7 [3–54]	0.07
Hospitalization (days)		18 [4–120]	17 [4–120]	29 [11–109]	**0.0002**
MELD score		15 [6–37]	15 [6–37]	14 [6–30]	0.72
Cause of TX					0.09
	AC	30 (26%)	25 (29.8%)	5 (16.1%)	
	AC+HCC	24 (20.7%)	15 (17.9%)	9 (29%)	
	HCC	26 (22.6%)	23 (27.4%)	3 (9.7%)	
	PBC	5 (4.34%)	3 (3.6%)	2 (6.5%)	
	FHF	6 (5.22%)	4 (4.8%)	2 (6.5%)	
	AMIL	5 (4.34%)	4 (4.8%)	1 (3.2%)	
	OTHERS	19 (13.4%)	10 (11.9%)	9 (29%)	
Rejection during first month		14 (12.1%)			
Rejection (2 years after TX)		21 (18.2%)			

Continuous values expressed as median [range]. Mann–Whitney U-test was used to compare differences for all continuous parameters studied, with the exception of cold ischemia, which was normally distributed (*t*-test); Bold: significant *p* values (*p* < 0.05). AC: alcoholic cirrhosis; HCC: hepatocellular carcinoma; PBC: Primary biliary cirrhosis; FHF: fulminant hepatic failure; AMIL: hepatic amyloidosis; MELD: Model for End-Stage Liver Disease.

**Table 2:** Comparison between patients with and without rejection.

	Stable (*n* = 101)	Rejection (*n* = 14)	*p*
Age (years)	57 [18–71]	48 [21–60]	**<0.0001**
Sex (male)	54 (53%)	8 (57.1%)	0.5
Donnor age (years)	62 [17–84]	64 [14–82]	0.84
Warm Ischemia (min)	32 [22–60]	30 [22–36]	0.24
Cold Ischemia (min)	385 [270–575]	380 [250–440]	0.31
Previous ICU	8 (7.8%)	3 (21.4%)	0.13
Previous ICU (days)	3 [1–58]	1 [1–34]	0.5
ICU post-TX (days)	6 [3–55]	6 [4–14]	0.89
Hospitalization (days)	17 [4–120]	31 [11–67]	**0.01**

Continuous values expressed as median [range]. Mann–Whitney U-test was used to compare differences for all continuous parameters studied, with the exception of cold ischemia, which was normally distributed (*t*-test); Bold: significant *p* values (*p* < 0.05).

**Table 3:** Correlation between cfDNA levels and liver function markers in the total population during evolution after TX, stable patients and patients suffering liver damage.

cfDNA	Total		Stable Patients		Patients with Liver Damage	
	CC	*p*	CC	*p*	CC	*p*
ALT	0.306 **	**<0.0001**	0.348 **	**<0.0001**	0.212 **	**<0.005**
AST	0.337 **	**<0.0001**	0.398 **	**<0.0001**	0.222 **	**0.01**
Gamma-GT	−0.065	0.129	−0.102 *	**<0.05**	−0.007	0.93
Bilirubin	0.009	0.8	0.004	0.938	−0.113	0.153

Spearman test Correlation coefficient (CC) with 95% (*) or 99% (**) CI; significant *p* value in bold. ALT: Alanine aminotransferase; AST: Aspartate aminotransferase; Gamma-GT: Gamma-glutamyltransferase.

## Data Availability

The datasets generated and analyzed during the current study are available from the corresponding author upon reasonable request. The data are not publicly available due to ethical reasons.

## Supplementary Materials

The following supporting information can be downloaded at: https://www.mdpi.com/article/10.3390/jcm14238400/s1. Figure S1: Alanine aminotransferase, aspartate aminotransferase, gamma-glutamyltransferase and bilirubin levels during the first 30 days; Table S1: Causes and time to death of transplanted patient.
