# Supplementary material for: Cell-Free DNA Levels During the First Hours After Liver Transplantation: A Key Biomarker for Patient Survival and Outcomes
Source: J Clin Med. 2025 Nov 27;14(23):8400. doi: 10.3390/jcm14238400 (PMC12693361; doi:10.3390/jcm14238400)
Supplement: Supplementary file 1 [file jcm-14-08400-s001.zip › jcm-3951976-supplementary.pdf]

Supplementary Table S1. Causes and time to death of transplanted patient

Nº patient	Time to rejection (days)	Time to death (months)	Causes of death
**65**	420	14	Graft dysfunction and multiple organ failure after ductopenic rejection and graft dysfunction
**125**	10	34	Respiratory failure due to pneumonia
**181**	-	4 days	Subarachnoid hemorrhage, familial amyloidotic polyneuropathy.
**227**	-	14	Multiple organ failure as a consequence of shock septic (*Candida albicans*)
**253**	-	3	Multiple organ failure as a consequence of shock septic (*cytomegalovirus* infection)
**278**	-	7	Multiple organ failure as a consequence of shock septic (intra-abdominal origin, *Pseudomonas aeruginosas*)
**286**	-	3	Long period at the ICU with severe complications. Multiple organ failure as a consequence of shock septic (abdominal origin, *Escherichia coli* and *Candida albicans*)
**308**	-	27	Respiratory failure due to Microcytic Lung Carcinoma progression
**319**	-	31	Liver failure due to autoimmune hepatitis
**384**	-	4	Pulmonary lymphoproliferative neoplasm
**406**	-	14	Multiple organ failure as a consequence of shock septic (*Aspergillus Niger*)

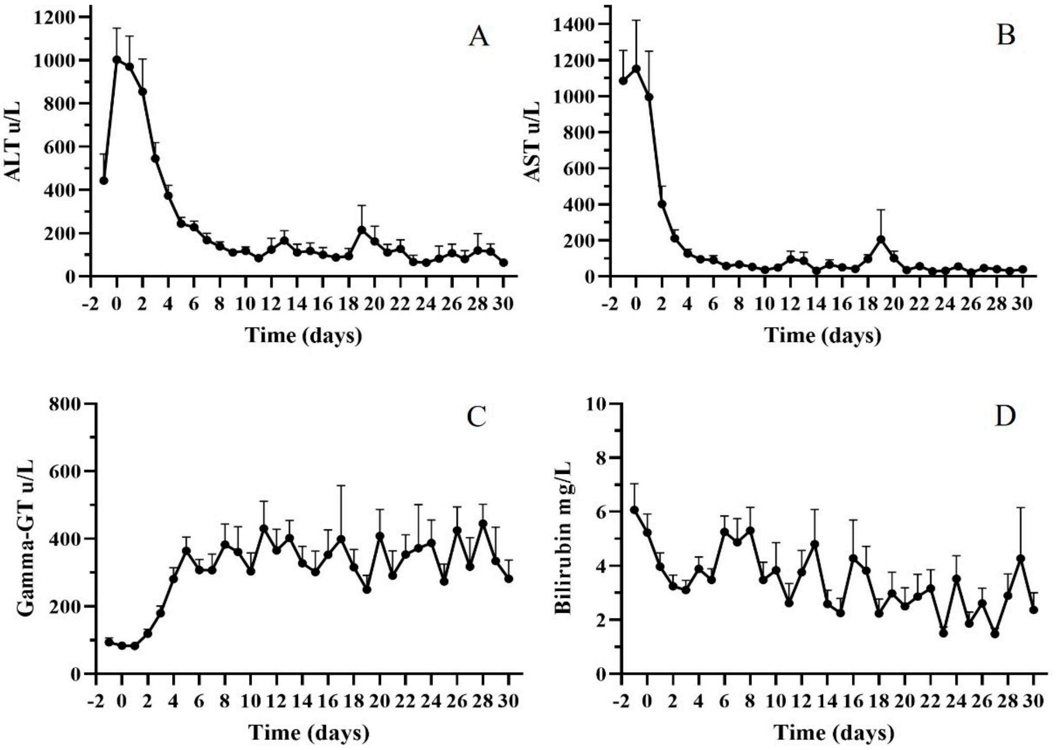

Supplementary Figure S1. A) alanine aminotransferase (ALT); B) aspartate aminotransferase (AST); C) gamma-glutamyltransferase (gamma-GT) and D) bilirubin levels during the first 30 days
